# A negative screen for mutations in calstabin 1 and 2 genes in patients with dilated cardiomyopathy

**DOI:** 10.1186/1477-5751-11-4

**Published:** 2012-01-11

**Authors:** Diogo G Biagi, José G Mill, Alfredo J Mansur, José E Krieger, Alexandre C Pereira

**Affiliations:** 1 Laboratory of Genetic and Molecular Cardiology, Heart Institute, University of Sao Paulo Medical School, Brazil; 2 Department of Physiology, Federal University of Espírito Santo, Vitória, Brazil; 3 Heart Institute, University of Sao Paulo Medical School, Brazil

**Keywords:** Genetic screening, Calstabin, FKBP12, FKBP12.6

## Abstract

**Background:**

Calstabins 1 and 2 bind to Ryanodine receptors regulating muscle excitation-contraction coupling. Mutations in Ryanodine receptors affecting their interaction with calstabins lead to different cardiac pathologies. Animal studies suggest the involvement of calstabins with dilated cardiomyopathy.

**Results:**

We tested the hypothesis that calstabins mutations may cause dilated cardiomyopathy in humans screening 186 patients with idiopathic dilated cardiomyopathy for genetic alterations in calstabins 1 and 2 genes (*FKBP12 *and *FKBP12.6)*. No missense variant was found. Five no-coding variations were found but not related to the disease.

**Conclusions:**

These data corroborate other studies suggesting that mutations in *FKBP12 *and *FKBP12.6 *genes are not commonly related to cardiac diseases.

## Background

Ryanodine receptors (RyRs) are the main proteins in the sarcoplasmic reticulum (SR) calcium-release channel and are modulated by other proteins in the regulation of muscle excitation-contraction coupling[1]. Calstabins 1 and 2 (*FKBP12 *and *FKBP12.6 *genes, respectively) bind both RyR1 (skeletal form) and RyR2 (cardiac form) although calstabin1 has higher affinity to RyR1, and calstabin2 to RyR2 [2-4]. The RyR-calstabin interaction occurs to stabilize the RyR channel in the closed state, but its specific amino acids binding site is still controversial [5-7]. Although, calstabin1 deficient mice display normal skeletal muscle phenotype, they have severe Dilated Cardiomyopathy (DCM) and ventricular septal defects [8]. Mice with a disruption in calstabin2 display hypertrophic cardiomyopathy only in males. However, both males and females present a similar dysregulation of Ca^2+ ^release [9].

Disease-causing mutations in RyRs 1 and 2 affecting the interaction with calstabins were described as leading to different phenotypes [1]. Wehrens and colleagues reported RyR2 mutations which decrease the affinity of RyR2 with calstabin2 in patients with Catecholaminergic Polymorphic Ventricular Tachycardia (CPVT) [7]. However, no mutation in calstabin2 seemed to be involved with the disease [10]. The association of calstabins with heart disease was also tested in a familial Hypertrophic Cardiomyopathy cohort, and no mutation was found in all 252 patients [11]. Recently, Oyama and colleagues have reported an increased expression of *FKBP12.6 *gene in Great Dane dogs with DCM [12]. These results, taken together with the dilated cardiomyopathy phenotype of calstabin1 knockout mice, suggest that calstabins may be associated with DCM development. Thus, the aim of the present study is to test the hypothesis that mutations in calstabins genes (*FKBP12 *and *FKBP12.6*) occur, and can cause human DCM phenotype.

## Results

Considering the 186 Idiopathic Dilated Cardiomyopathy (IDC) patients screened, 75% were male and 25% female. The mean age was 44.8 ± 12.4. The population had the following ethnic distribution: 14.5% blacks, 15.5% mullatos and 69.8% whites.

No missense mutation was found in *FKBP12 *nor in *FKBP12.6*. Five new allelic variants were found (Table 1). Two of them were found in the control group (age and ethnic matched) with no significant difference in allelic nor genotypic frequency. Three allelic variants, one in the promoter region and two in the Exon1 5'UTR of *FKBP12*, were not present in the control group. Bioinformatics data suggest that these allelic variants have a very low or none probability to be pathological. From all SNPs found in our population, only one SNP with minor allelic frequency (MAF) not available in dbSNP was found (Table 2). All the alterations in cases and controls met the Hardy-Weinberg equilibrium.

**Table 1:** New allelic variants found in *FKBP12 *and *FKBP12.6 *gene.

Gene	Site	MAF: cases (n = 186)	Reference (coding DNA)	MAF: controls (n = 288)	p value	Risk by bioinformatics analyses
*FKBP12*	Promoter	0.0026	-40C>A	0.0052	0.25	-
*FKBP12*	Promoter	0.0026	-7G>A	0	-	Low
*FKBP12*	Exon 1 - 5' UTR	0.0026	c.-72C>G	0	-	None
*FKBP12*	Exon 1 - 5' UTR	0.0026	c.-65C>G	0	-	None
*FKBP12.6*	Exon 4 - 3'UTR	0.0026	c.*26G>A	0.0017	0.55	-

MAF: Minor Allele Frequency

**Table 2:** SNPs found in *FKBP12 *and *FKBP12.6 *gene in cases.

Gene	Site	SNP	Troca	MAF: cases (n = 186)	MAF: dbSNP (YRI - CEU)
*FKBP12*	Intron 2	rs6074549	C/G	0.0026	0.008 - 0

*FKBP12.6*	Exon 1 - 5' UTR	rs116520786	C/T	0.14	0.25 - 0
	Exon 1 - 5' UTR	rs111634163	C/T	0.06	NA
	Intron 3	rs13016301	A/T	0.07	0 - 0.18
	Intron 3	rs1424344	C/T	0.18	0.32 - 0
	Exon 4 - 3'UTR	rs14388	C/T	0.17	0.4 - 0.008
	Exon 4 - 3'UTR	rs114658911	C/T	0.005	0 - 0.005

MAF: Minor Allele Frequency; NA: Not Available; CEU: Northern and Western Europe; YRI: Yoruba in Ibadan, Nigeria

## Discussion

Data from animal models point out FKBPs as important candidate genes for cardiac diseases. In fact, several mutations in RyR1 and RyR2 proteins cause decreased affinity between RyRs and calstabins, leading to abnormal calcium cycling, a major hallmark of several cardiac phenotypes, including heart failure. Mutations affecting RyR-calstabin interactions are well described for some diseases such as: Left Ventricule Noncompaction (LVNC), Central Core Disease (CCD) and Catecholaminergic Polymorphic Ventricular Tachycardia (CPVT). However, little is known if mutations in calstabins could lead to disease by themselves. Genetic analyses of *FKBP12 *were done only in two cohorts of diseases (LVNC (48 patients) [13] and CCD (27 patients) [14]) and in one family with LVNC. No mutation was found in any of this studies. Recently, genetic analyses of *FKBP12.6 *have been done in 16 patients with CPVT and in 232 patients with hypertrophic cardiomyopathy and, also, no mutations were found.

To our knowledge, this study has been the first report of calstabins 1 and 2 genetic screening in idiopathic DCM patients. We found five new allelic variations and none of them caused amino acid change. The alterations c.*26G>A in *FKBP12.6 *gene and -40C>A in *FKBP12 *gene were also found in the control population. For the three other alterations we measured the risk of being disease-causing by the analyses of three different bioinformatics software, concerning their possible involvement in splicing, microRNA and transcription factor binding sites [15-17]. The -7G>A alteration in the promoter region of *FKBP12 *was predicted as low risk, although its create a new biding site for the NRF2 transcription factor (TF). NRF2 enhances gene expression in response to oxidative stress, which is involved in the development and progression of heart failure [18,19]. This suggests that the -7G>A alteration could result in overexpression of *FKBP12 *gene. Although overexpression of *FKBP12 *gene has been described modulating RyR2 in rabbit myocytes [20], we believe that this mutation alone could not be responsible for disease development, once the concentration of calstabin1 in the heart is naturally much higher than calstabin2 and does not hinder calstabin2 interaction with RyR2 [4]. Further studies are needed to assess the impact of the overexpression of calstabins1 in human cardiomyocytes. The c.-72C>G and c.-65C>G alterations in the exon 1 5'UTR region were predicted with none risk to be related with the disease. Overall, we did not find any alterations possibly related with the disease.

We found 7 silent SNPs with MAFs ranging between MAFs of European and African populations, which are the main founders of Brazilian population. Among the SNPs presented in the sequenced regions with allelic frequency described on dbSNP, only one was not found in our population (data not shown- rs77743612). This may be explained by its low MAF in the Yoruba population (0.014) and absence in the CEU population. Since these silent SNPs were not overrepresented in our population, it is quite improbable that they might be related with the disease.

Today, mutations in about 35 genes are described in idiopathic DCM. Together, they do not explain more than 10% of cases, indicating that more genes might be involved with the disease pathogenesis. Animal models suggested that calstabin proteins might be associated with DCM. Our result, taken together with other calstabin genetic screening efforts, leads us to believe that mutations in *FKBP12 *and *FKBP12.6 *genes are not commonly related to cardiac diseases.

## Conclusion

Calstabin 1 and 2 are not involved in idiopathic DCM development in Brazilian patients.

## Methods

### Study population

One-hundred-eighty-six (186) patients with Idiopathic Dilated Cardiomyopathy (IDC) were selected from two different heart failure cohorts from the Heart Institute (InCor), University of São Paulo Medical School. In both groups, the diagnosis of heart failure was made according to previously published criteria [21,22]. The heart failure etiology classification followed previous recommendations[22]. As such, the diagnosis of chronic heart failure was made through both clinical and imaging procedures when necessary. Ischemic cardiomyopathy diagnosis was made when a clear history of previous myocardial infarction and no other probable causes of heart dysfunction were present or, alternatively, through coronary angiography. All patients with the final diagnosis of idiopathic dilated cardiomyopathy were studied through coronary angiography to exclude the diagnosis of ischemic cardiomyopathy. Table 3 shows clinical and functional characteristics of the IDC Cohort.

**Table 3:** Clinical and functional characteristics of IDC Cohort

IDC Cohort	
**Clinical**	
Biochemical	
Hemoglobin (mg/dL)	12.7 ± 1.97
Total Cholesterol (mg/dL)	185 ± 55
Triglycerides (mg/dL)	106 ± 56
HDL (mg/dL)	45.7 ± 17.3
LDL (mg/dL)	116 ± 44
Creatinin (mg/dL)	1.2 ± 0.3
BMI (kg/m^2^)	25.08 ± 4.65
Heart Rate (pm)	76.35 ± 13.57
Diastolic Blood Pressure (mmHg)	67.41 ± 14.56
Systolic Blood Pressure (mmHg)	104.44 ± 18.9
**Echocardiographic**	
Left Atrium Diameter, mm	47.52 ± 8.48
Aortic Diameter, mm	32.57 ± 4.26
Right Ventricle Diameter, mm	27.19 ± 6.14
Left Ventricle	
Intraventricular Septum, mm	8.39 ± 1.48
Posterior Wall Thickness, mm	8.31 ± 1.21
Diastolic Diameter, mm	73.55 ± 10.06
Systolic Diameter, mm	64.01 ± 9.58
Ejection Fraction, (%)	35.08 ± 8.9
Mass, g	268.74 ± 77

### Molecular Genetic Analyses

Extraction of genomic DNA was performed from leukocytes separated from whole blood using a standard salting-out method [23]. DNA samples were further diluted with PCR grade water to a concentration of 10 ng/μL. All coding exons and flanking sequences of *FKBP12 *(reference sequence: NM_000801.3) and *FKBP12.6 *(reference sequence: NM_004116.3) were analyzed for mutations, with at least 20 nucleotides into introns. Intronic primers were designed using the software Primer3 [24]. The final optimal reaction conditions were empirically determined; primer sequences are described in Table 4, and PCR conditions are available upon request. Samples were pre-selected for bidirectional sequencing by High-Resolution Melting (HRM) curves manually analyzed or were bidirectionally sequenced directly. For HRM, the reaction mixture used BioTaq DNA Polymerase (BioQuimica, Brazil) and consisted of 10 ng of genomic DNA, 1× Assay buffer, 2 mM MgCl2, 200 nM of each primer, 200 μM of dNTPs, 1,5 μM of SYTO9 (Invitrogen, Carlsbad, USA), 0.5 U of BioTaq DNA Polymerase and PCR grade water in a volume of 10 μL. PCR cycling and HRM analysis were performed on the Rotor-Gene™ 6000 equipment (Qiagen, Courtaboeuf, France). Bidirectional sequencing was performed by 3500xL Genetic Analyzer (Applied Biosystems, EUA) using BigDye^® ^Terminator v3.1 Cycle Sequencing Kit (Applied Biosystems, EUA) and manufacturer protocols. The frequency of all identified variants was determined in 288 age and ethnic matched controls by HRM.

**Table 4:** Primers for *FKBP12 *and *FKBP12.6 *amplification of coding exons and flanking region sequences.

Gene	Exon	Primer	Amplicon
*FKBP12*	1F	5'- TCTCGGGGCTTCTGGGCTTC -3'	269
		
	1R	5'- GCGCCACTACTCACCGTCTC -3'	
	
	2F	5'- CATGCCCGTCTCTGTCTCCTCA -3'	242
		
	2R	5'- AAGCCCCAGGCTGCCCCTGA -3'	
	
	3F	5'- TGCTGCTGGTTTCTGACTTGT -3'	299
		
	3R	5'- TGGCATGAGGGTGAGACTTTTC -3'	
	
	4F	5'- TTTCTTACAGAGACAGTTGGGG -3'	253
		
	4R	5'- GCCCTTCAGTATTCCATTTCCT -3'	

*FKBP12.6*	1F	5'- GAATGGATGGATGGAGGATG -3'	527
		
	1R	5'- TCCTAGACCCCCAAGAATCC -3'	
	
	2F	5'-CCAGAGAGGTGACGTGA-3'	390
		
	2R	5'-TAGACTGGCTGGGAGGA-3'	
	
	3F	5'-GGGAGTTTACTCATGACCA-3'	368
		
	3R	5'-CAGGGACACAAAAGAGGTA-3'	
	
	4F	5'-CAGTCACATCTCTGCTGA-3'	510
		
	4R	5'-CGCATGAACACTGACGAA-3'	

The importance of variants out of coding regions that were not present in the control sample was analyzed *in silico *by FastSNP[17], MutationTaster [15] and miTarget [16]; concerning the transcription factor binding site, splicing and miRNA binding sites, respectively.

### Statistical analysis

Data are presented as mean ± standard deviation (SD) for continuous variables and as frequencies for categorical variables. Statistical analyses were performed with SPSS software 13.0. Hardy-Weinberg equilibrium was evaluated by a Chi-square test. Allelic frequencies comparison was evaluated Fisher's exact test.

### Ethics Statement

The investigation conforms to the principles outlined in the Declaration of Helsinki and the study protocol was approved by the Ethics Committee for Medical Research on Human Beings of the *Hospital das Clínicas *from University of São Paulo Medical School. Signed informed consent was obtained from all participants from both samples.

## List of abbreviations

A: Adenine; C: Cytosine; CCD: Central Core Disease; CPVT: Catecholaminergic Polymorphic Ventricular Tachycardia; DCM: Dilated Cardiomyopathy; G: Guanine; IDC: Idiopathic Dilated Cardiomyopathy; LVNC: Left Ventricule Noncompaction; MAF: Minor Allelic Frequency; RyRs: Ryanodine Receptors; SNP: Single Nucleotide Polymorphism; SR: Sarcoplasmic Reticulum; T: Thymine; TF: Transcription Factor; UTR: Untranslated region.

## Competing interests

The authors declare that they have no competing interests.

## Authors' contributions

DGB participated in the design of the study, carried out the molecular genetic studies, analyses and drafted the manuscript. AJM coordinated the creation of the studied population. JGM coordinated the creation of the control population. JEK participated in the design of the study. ACP participated in the design and coordination of the study and helped to draft the manuscript. All authors read and approved the final manuscript.
